# Does Cannabis Intake Protect Against Non-alcoholic Fatty Liver Disease? A Two-Sample Mendelian Randomization Study

**DOI:** 10.3389/fgene.2020.00949

**Published:** 2020-08-14

**Authors:** Xiaokun Wang, Zhipeng Liu, Wanqing Liu

**Affiliations:** ^1^Department of Pharmaceutical Sciences, Eugene Applebaum College of Pharmacy and Health Sciences, Wayne State University, Detroit, MI, United States; ^2^Department of Medicinal Chemistry and Molecular Pharmacology, College of Pharmacy, Purdue University, West Lafayette, IN, United States; ^3^Department of Pharmacology, School of Medicine, Wayne State University, Detroit, MI, United States

**Keywords:** cannabis, marijuana, non-alcoholic fatty liver disease, Mendelian randomization, GWAS

## Abstract

**Background and Aim:**

Non-alcoholic fatty liver disease (NAFLD) is the most common chronic liver disease. Previous observational studies suggested that cannabis use may be associated with a lower risk for NAFLD but the causal relationship remains unclear. We aim in this study to examine the causal effect of cannabis consumption on the risk of NAFLD using a Mendelian randomization analysis. Clarifying this causal effect is important for cannabis-based drug discovery for NAFLD.

**Methods:**

We used data from the largest-to-date GWAS meta-analysis on lifetime use of cannabis (yes or no) consisting of three cohorts [International Cannabis Consortium (ICC), 23andMe, and the UK Biobank] of European ancestry (total *N* = 184,765). We also used other GWAS data on cannabis use dependence and cannabis use disorder (CUD). The NAFLD GWAS data were generated from the UK Biobank population (1,122 cases and 399,900 controls). The inverse variance weighted (IVW) method was used to assess the causal impact of cannabis lifetime use on the risk of NAFLD. We also performed a sensitivity analysis using weighted median estimator and MR-Egger.

**Results:**

There was no statistically significant causal effect between either the lifetime cannabis use, cannabis use dependence or CUD and the risk for NAFLD (*p* > 0.05 for all tests). No significant pleotropic effect was observed based on both MR-PRESSO global test (*p* = 0.99) and the modified Q’ statistics. However, the study may be underpowered.

**Conclusion:**

Our results demonstrated no evidence that cannabis consumption has a causal effect of protection against the development of NAFLD.

## Introduction

Chronic liver disease is a serious health problem, the morbidity and mortality are steadily increasing over the years. Non-alcoholic fatty liver disease (NAFLD) represents the most common form of chronic liver disease, encompassing a spectrum from simple hepatic steatosis to steatohepatitis (NASH) with variable degrees of fibrosis. The global prevalence of NAFLD is currently estimated to be 25%, including both adults and children (Younossi et al., 2016). In the United States, NAFLD affects up to 100 million Americans (Spengler and Loomba, 2015). However, there are no approved effective pharmacologic agents for NAFLD treatment or prevention. Clarifying the factors that can reduce the risk of NAFLD or slow the progression of fatty liver disease is of importance to alleviating the global medical and economic burden, as well as to provide clues for new drug discovery and development.

Cannabis, also known as marijuana, is the most widely used but strictly regulated illicit drug made from *cannabis sativa*. According to an official statistical report from the United Nations in 2015, about 2.7–4.9% of world inhabitants who recently have used cannabis. In the clinic, cannabis is mainly used for alleviating certain types of chronic pain, including pain from nerve damage, cancer, inflammation, and spasticity. Increasing evidence has shown that cannabis intake is associated with a lower risk of NAFLD (Adejumo et al., 2017; Kim et al., 2017; Farooqui et al., 2019). However, the causal relationship between cannabis consumption and the NAFLD risk in humans remains unclear.

Mendelian randomization (MR) is a strategy for examining causal relationships between an exposure (such as cannabis use) and outcome (such as NAFLD) free from confounding or reverse causality bias in observational or epidemiological studies. It uses genetic variants that associate with the exposure to analyze the causality between the exposure and outcome in situations where randomized controlled trials are not possible or unethical. In this study, we used single nucleotide polymorphisms (SNPs) significantly associated with lifetime cannabis use or cannabis use dependence reported in some recent genome-wide association studies (GWASs) as an instrument to clarify the causation of the cannabis consumption and the risk for NAFLD. Our analysis revealed no evidence supporting a causal impact of cannabis intake on reducing the risk for the development of NAFLD.

## Materials and Methods

### GWAS Summary Data for Lifetime Cannabis Use (Cannabis Consumption or Not)

The associations of genetic variants with the lifetime cannabis use were taken from the largest-to-date GWAS meta-analysis consisting of three cohorts [International Cannabis Consortium (ICC), 23andMe, and the UK Biobank] of European ancestry (total *N* = 184,765). The phenotype is characterized as a binary variable indicating the self-reported use of cannabis during lifetime (yes or no). On average, 42.8% of the individuals from ICC, 43.2% of the individuals from 23andMe, and 22.3% of the individuals in the UK Biobank cohort had revealed the use of cannabis during their lifetime. Details regarding the GWAS meta-analysis and ethical approval can be found in the original publications (Pasman et al., 2018).

### GWAS Summary Data for Cannabis Use Disorder

The genetic variant rs56372821 (OR = 0.803, *p* = 9.09 × 10^–12^) associated with cannabis use disorder (CUD) was selected from the result of a GWAS meta-analysis based on the largest cohorts of diagnosed CUD reported so far (Demontis et al., 2019). The samples include (1) a Danish nationwide population-based cohort consisting of 2,387 individuals with a diagnosis of CUD and 48,985 controls which were collected by the Lundbeck Foundation Initiative for Integrative Psychiatric Research (iPSYCH), and (2) Icelandic samples consisting of 5,501 cases with CUD and 301,041 controls (the deCODE cohort). The CUD was defined as a problematic and persistent use of cannabis based on an International Statistical Classification of Diseases and Related Health Problem, 10^*th*^ revision (ICD-10 F12.1-12.9). Detailed information can be found in the original publications (Demontis et al., 2019).

### GWAS Summary Data for Cannabis Dependence

The instrumental variant rs1409568 (β = −0.50, *p* = 3.95 × 10^–8^) reported to be associated with cannabis use dependence is based on the result of a meta-analysis of GWAS data on 2,080 DSM-IV cannabis dependence cases and 6,435 cannabis exposed controls of European–American descent (Agrawal et al., 2018). Cannabis dependence cases was defined as the individuals who met criteria for DSM-IV cannabis dependence. The cases who reported a lifetime history of cannabis exposure but did not meet criteria for DSM-IV cannabis dependence were characterized as controls. Detailed information was described in related publications (Agrawal et al., 2018).

### GWAS Summary Data for Cannabis Dependence Severity

The GWAS meta-analysis for DSM-IV cannabis dependence criterion count included 3 cohorts (the Yale-Penn Study, Study of Addiction: Genetics and Environment [SAGE], and International Consortium on the Genetics of Heroin Dependence [ICGHD]) (Sherva et al., 2016). The participants included 4,456 cases and 10,298 controls consisting of 6,000 African–American and 8,754 European–American. The phenotype was characterized as criterion counts for cannabis use dependence. Detailed information can be found in the original publication (Sherva et al., 2016).

### GWAS Summary Data for NAFLD

Since the summary level GWAS data for NAFLD is not publicly available, we first performed a GWAS analysis for NAFLD using the UK Biobank samples (*N* = 1,122 cases and 399,900 controls). As reported in our previous study (Liu et al., 2020), NAFLD was defined as a binary phenotype based on the ICD code [ICD-9 571.8 “Other chronic non-alcoholic liver disease” and ICD-10 K76.0 “Fatty (change of) liver, not elsewhere classified”]. We excluded individuals with hepatitis B or C infection or other liver diseases in our analysis. The associations between NAFLD and SNPs were analyzed using SAIGE with sex, birth year, and the first four genetic principal components as covariates.

### Genetic Instruments Construction

For lifetime cannabis use (yes or no), we constructed two genetic instruments with different threshold of significance. The first one includes five independent genome-wide significant SNPs (*p* < 5E-08, pairwise *R*^2^ < 0.001 based on the European subset of the 1000 Genomes Project Phase 3 data). The second one consists of independent variants with a more liberal threshold (*p* < 1E-05, *R*^2^ < 0.001, 67 SNPs). The SNPs used for MR analysis were described in Supplementary Table 1.

For CUD, the single SNP rs56372821 (*p* = 9.09 × 10^–12^) was used as the genetic instruments. For cannabis dependence, the single SNP rs1409568 (*p* = 2.9 × 10^–7^) was used as the instrument variable. For cannabis dependence severity with two genome-wide significant SNPs, rs143244591 and rs77378271 (*p* < 5E-08), we treated them as both independent instrument variables and a combined instrument variable. For the SNPs associated with cannabis dependence in Sherva et al. (2016) where both African–American and European–American samples were used, summary data (p, beta and se) are only available for the combined sample set. We therefore used this combined summary data for our MR analysis.

The strengths of the instrumental variables were evaluated by the *F* statistics = $\left(\frac{n-k-1}{k}\right)\,\left(\frac{R^{2}}{1-R^{2}}\right)$, in which n is the sample size, k is the number of SNPs, and *R*^2^ is the proportion of interindividual variance in lifetime cannabis use explained by the instrument. The variance explained (*R*^2^) by each SNP was estimated as $\frac{2*{\hat{\beta }}^{2}*M\,A\,F*\left(1-M\,A\,F\right)}{2*{\hat{\beta }}^{2}*M\,A\,F*\left(1-M\,A\,F\right)+{\left(s\,e\,\left(\hat{\beta }\right)\right)}^{2}*2*n*M\,A\,F*\left(1-M\,A\,F\right)}$, in which $\hat{\beta }$ is the coefficient of the association between the SNP and phenotype, $s\,e\,\left(\hat{\beta }\right)$ is the standard error of the coefficient, and MAF is the minor allele frequency (Teslovich et al., 2010). We summed up the *R*^2^ of all the SNPs in an instrument for the calculation of F statistics due to the independence of the genetic variants. The variance explained by the two genetic instruments were 0.11% (*n* = 5) and 0.86% (*n* = 67), respectively. The F statistics of the two genetic instruments for lifetime cannabis use were 39 and 24, respectively. The F statistics of the genetic instruments for CUD, cannabis dependence and cannabis dependence criterion count were 47, 31, and 35, respectively. The strengths of all instruments were stronger than the empirical threshold of 10 (Douglas Staiger, 1997).

### MR Estimation

We used the inverse variance weighted (IVW) (Burgess et al., 2013) method to estimate the causal effect of lifetime cannabis use on the NAFLD risk using the two genetic instruments, respectively. Assuming that the MR assumptions are met, the IVW method provides the most accurate causal estimation (Bowden et al., 2016b). Besides, we performed a sensitivity analysis using two other methods [weighted median estimator (Bowden et al., 2016a) and MR-Egger (Bowden et al., 2015)] that are more robust in the existence of horizontal pleiotropy. The weighted median estimator is unbiased given that more than 50% of the SNPs are valid instruments. MR-Egger estimation is consistent with the causal effect assuming that the genetic instrument strength is independent of the pleiotropic effects. This is a relaxation of the no pleiotropy assumption. Furthermore, we assessed the existence of the potential heterogeneity and pleiotropy using the Q’ statistics with modified second-order weights (Bowden et al., 2018a) and the MR-PRESSO global test (Verbanck et al., 2018), respectively. If the MR-PRESSO global test is significant (*p* < 0.05), the causal effect is estimated again after removing the SNPs identified as pleiotropic outliers. We considered the causal effect is significant if the *p*-value of the IVW estimation is less than 0.05, the directions of the three estimates (IVW, weighted median, and MR-Egger) are consistent, and no significant heterogeneity and pleiotropy identified by the Q’ statistics and the MR-PRESSO global test. The Wald ratio method was used for calculating causal estimates with a single SNP (Haycock et al., 2016). The power of the MR estimates were calculated using the online power calculator^1^ by Stephen Burgess (Burgess, 2014). All the analyses and visualizations were performed using R v.3.5.0^2^ with the “MendelianRandomization” (Yavorska and Burgess, 2017), “MRPRESSO” (Verbanck et al., 2018), and “RadialMR” (Bowden et al., 2018b) packages.

## Results

### Lifetime Cannabis Use Has No Significant Causal Effect on the Risk for NAFLD

To explore the causal relationship between lifetime cannabis use and NAFLD, we performed the MR analysis using five independent genome-wide significant SNPs (*p* < 5E-08) as the genetic instrument for lifetime cannabis use. These five independent genome-wide significant SNPs are located in *CADM2*, *NCAM1*, *ATP2A1*, *ZNF704*, and *SMG6*, which are associated with lifetime cannabis use (yes or no), were used as genetic instruments under the *p*-value threshold 5E-08. The *CADM2* gene encodes a member of the synaptic cell adhesion molecules 1 family, and is associated with a range of behavioral and metabolic traits, such as educational attainment, alcohol and obesity (Morris et al., 2019). *NCAM1* (Neural Cell Adhesion Molecule 1) belongs to the immunoglobin superfamily, and has been considered a susceptibility gene for schizophrenia and bipolar disorder (Chen et al., 2012). The *ATP2A1* gene encodes one of the sarcoplasmic/endoplasmic reticulum Ca^2+^ ATPase, which can catalyze the ATP-dependent transport of Ca^2+^ from the cytosol to the sarcoplasmic reticulum lumen (Bruels et al., 2019). *ZNF704* is a zinc finger protein and might be related with cancer (Cheng et al., 2019). *SMG6* encodes a component of the telomerase ribonucleoprotein complex, and plays vital roles in the replication and maintenance of chromosome ends, as well as providing the endonuclease activity in the nonsense-mediated mRNA decay pathway.

**FIGURE 1:**
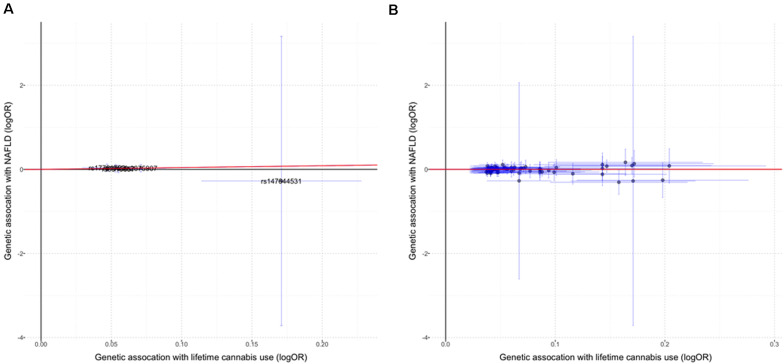
The causal effect of lifetime cannabis use on NAFLD risk. **(A)** MR estimation using the genetic instrument including five SNPs (*p* < 5E-08). **(B)** MR estimation using the genetic instrument including 67 SNPs (*p* < 1E-05). Red lines indicate the IVW (inverse variance weighted) estimates. Horizontal lines represent 95% confidence intervals for the associations with the exposure (cannabis use), while the vertical lines represent 95% confidence intervals for the associations with the outcome (NAFLD).

We did not observe significant causal effect of lifetime cannabis use on the risk for NAFLD (OR: 1.55; 95% CI: [0.72, 3.31], *p* = 0.26, Table 1 and Figure 1A). MR estimates using two orthogonal methods including weighted median and MR-Egger produced similar results. The estimation is not likely to be biased by the pleiotropic effect as tested by the MR-PRESSO global test (*p* = 0.99) and the modified Q’ statistics (*p* = 0.99). To achieve more statistical power in the MR analysis, we then expanded the list of the genetic variants by including 67 independent SNPs with *p*-value less than 1E-05. Similar to the analysis using 5 genome-wide significant SNPs, the causal effect of lifetime cannabis use on NAFLD risk remains not significant (OR: 1.02; 95% CI: [0.80, 1.30], *p* = 0.86, Table 1 and Figure 1B).

**TABLE 1:** Causal effect of lifetime cannabis use on NAFLD risk.

**Instrument**	**R∧2^#^**	***F* statistics**	**IVW(inverse variance weighted)**		**Weighted median**		**MR-Egger**		**Pleiotropy test**	
						
			**OR (95% CI)**	***p***	**OR (95% CI)**	***p***	**OR (95% CI)**	***p***	**MR-PRESSO global test *p***	**Modified Q’ *p***
5 SNPs (*p* < 5E-08)	0.11	39	1.55 (0.72, 3.31)	0.26	1.54 (0.64, 3.75)	0.34	0.83 (0.0066, 104.79)	0.94	0.99	0.99
67 SNPs (*p* < 1E-05)	0.86	24	1.02 (0.80, 1.30)	0.86	1.20 (0.85, 1.69)	0.31	1.15 (0.64, 2.08)	0.64	0.81	0.85

*R∧2^#^: percentage of the variance explained.*

### Cannabis Dependence Has No Significant Causal Effect on the Risk for NAFLD

While the lifetime cannabis may not be able to reflect the amount and frequency of cannabis consumption, we further set out to test the causal relationship between more often use of cannabis and the NAFLD risk. Three GWASs of problematic use or diagnosed CUD has reported genome-wide significant independent risk loci (Sherva et al., 2016; Agrawal et al., 2018; Demontis et al., 2019). We used the results from these GWASs to estimate the causal association of cannabis dependence and risk of NAFLD. The genetic variant rs56372821 associated with CUD is also a strong expression quantitative trait locus (eQTL) for cholinergic receptor nicotinic α2 subunit (*CHRNA2*) (Demontis et al., 2019). The SNP rs1409568 associated with cannabis dependence were also demonstrated to potentially play a role of an enhancer in addiction-relevant brain regions (Agrawal et al., 2018). Both of these two instrumental variables demonstrated no significant causal association with the risk for NAFLD (rs1409568, OR: 1.40; 95% CI: [0.98, 1.99], *p* = 0.063; rs56372821, OR: 1.16; 95% CI: [0.70, 1.95], *p* = 0.56) (Table 2). In addition, three independent SNPs rs143244591, rs146091982, and rs77378271 were reported to be associated with cannabis dependence based on the *DSM-IV* criteria (Sherva et al., 2016). The variant rs146091982 was excluded from this study since it is not available in the NAFLD GWAS data. The variant rs77378271 is an intronic SNP located in the CUB and Sushi multiple domains 1 gene (*CSMD1*), and rs143244591 is located about 10 kb upstream of the transmembrane 4 L six family member 18 gene (*TM4SF18*). Using these two SNPs as either separate or combined instrument variables, we did not observe any causal association between this cannabis dependence phenotype and the risk of NAFLD (IVW methods, OR: 0.84; 95% CI: [0.46, 1.51], *p* = 0.56) (Table 2). Taken together, our results demonstrated no significant causal association between cannabis use dependence and the risk of NAFLD.

**TABLE 2:** Causal effect of cannabis dependence on the risk of NAFLD.

**Instrument**	**Phenotype**	**GWAS**	**Sample size**	**Participants**	**R∧2^#^**	***F* statistics**	**IVW**		**Wald ratio**	
								
							**OR(95% CI)**	***p***	**OR(95% CI)**	***p***
rs56372821	Cannabis use disorder	Demontis et al., 2019	7888 cases and 350026 controls	European	0.0001	47	NA	NA	1.16 (0.70, 1.95)	0.56
rs1409568	Cannabis dependence vs. cannabis exposed controls	Agrawal et al., 2018	2080 cases and 6435 control	EA	0.004	31	NA	NA	1.40 (0.98, 1.99)	0.063
rs143244591 rs77378271	Cannabis criterion count	Sherva et al., 2016	4456 cases and 10298 control	EA and AA	0.005	35	0.84(0.46, 1.51)	0.558	1.11 (0.29, 4.32) 0.78 (0.41, 1.51)	0.875 0.467

*EA, European–American; AA, African–American; R∧2^#^, percentage of the variance explained.*

### Power Analysis

We further estimated the power of the MR analysis. For the lifetime cannabis use, with the 5-SNPs predictor, the power of detecting the observational odds ratio of 0.71 at the significance level of 0.05 was 5.7%. The maximum odds ratio needed to achieve 80% power is 0.08. With the 67-SNPs predictor, we have a limited power of 18.5% to detect the observational odds ratio of 0.71 at the significance level of 0.05. The maximum odds ratio needed to achieve 80% power is 0.41 (Ebrahim and Davey Smith, 2008; Kim et al., 2017). For CUD/dependence, the maximum odds ratios needed to achieve 80% power at the significance level of 0.05 were 0.00023 (cannabis use disorder), 0.27 (cannabis dependence) and 0.31 (cannabis criterion count), respectively. Overall, although we used the largest-to-date datasets, our study was significantly underpowered.

## Discussion

Currently, there are about 36.7 million active cannabis users in North America (Anthony et al., 2017). The legalization of cannabis in some states resulted in a gradual rise in the prevalence of cannabis use (Cerda et al., 2012). Over the past decades, emerging data have suggested an inverse association between cannabis use and the risk for NAFLD. Further clarifying whether or not cannabis use causally reduce the risk of NAFLD may validate a new drug discovery strategy for NAFLD treatment, e.g., via targeting the cannabinoid receptors. However, it remains a challenge to verify this causal relationship in humans due to the influence of confounding factors as well as the unethical design of clinical trials. In the present study, we performed an MR analysis by using SNPs that are reported to associate with lifetime cannabis use or cannabis use dependence to explore the causality of cannabis use for the risk of NAFLD. The selection of these SNPs which are used as instruments in this study is based on the results of the largest GWAS study (*n* = 184,765) about lifetime cannabis use up to date (Pasman et al., 2018), as well as three GWAS studies about cannabis problematic use (Sherva et al., 2016; Agrawal et al., 2018; Demontis et al., 2019). Our findings do not demonstrate a causal protective impact of cannabis consumption against the development of NAFLD. However, our study may be underpowered to confirm this hypothesis.

This is the first study to utilize the MR method to demonstrate that there is no evidence of the causal association between cannabis use and the risk of NAFLD, albeit that it is inconsistent with the previous observational and epidemiological studies. For instance, Kim et al. (2017) showed that cannabis use exerts a protective effect against NAFLD. Adejumo et al. (2017) demonstrated that cannabis use was associated with a lower prevalence of NAFLD. A meta-analysis also showed cannabis use reduced the prevalence of NAFLD (Farooqui et al., 2019). However, the majority of these studies are cross-sectional and retrospective observational analyses, without an examination for causality. Even after the adjustment of many factors in their statistical analysis, the data would still be possibly affected by residual confounding variables, such as insulin resistance, obesity, and other metabolic characteristics, etc. In a recent longitudinal study, it was reported that cannabis consumption produced a protective effect against liver steatosis in psychosis patients (Vazquez-Bourgon et al., 2019). However, this could be also confounded, e.g., whether frequent cannabis use would alter antipsychotic-induced metabolic change. On the other hand, cannabis use may reduce other risk factors for NAFLD thereby indirectly reduces the NAFLD risk. It is well-known that weight gain and increased BMI are one of the most important risk factors inducing the development of NAFLD. Recent studies in our lab and others have demonstrated that increased BMI or obesity is a causal factor for NAFLD (Stender et al., 2017). However, cannabis use is causally associated with reduced BMI (Pasman et al., 2018). It remains a key question of what is the mechanism underlying the negative association between cannabis use and NAFLD risk.

One of the limitations of the MR study is that pleiotropy may interfere with the results. In this study, we found no evidence of horizontal pleiotropy. To reduce the potential bias of our calculation, we also performed MR estimates using the genetic variants that were reported to be associated with problematic or persistent use of cannabis in three other GWASs. Collectively, no significant causal effect of cannabis use or dependence on the risk of NAFLD was observed. However, our study may be significantly limited by the low power. Although we used the largest-to-date GWAS data, the power might still be limited as indicated in our power calculation. This may be due to the potentially low heritability of the cannabis use phenotypes. Therefore, our study does not sufficiently exclude the possibility of the causal relationship between chronic cannabis use and the development of NAFLD. Further carefully designed studies using independent large-scaled populations and better or alternatively defined phenotypes are needed. Also, the “fatty liver disease” phenotype as presented in the UK biobank is a medical record-based trait, lacking a strict clinical diagnosis. Although we tried the best to remove other confounding factors, e.g., hepatitis B and C, etc., it may still not represent a carefully defined typical NAFLD phenotype. However, the SNPs that are significantly associated with this trait in our GWAS are very similar to what reported in previous GWASs for clinically defined NAFLD, e.g., PNPLA3 and TM6SF2 variants are the most significant ones (Liu et al., 2020), suggesting that the statistical estimation on high-risk variants for fatty liver disease is reliable. Nevertheless, future studies should further confirm this finding using GWAS data on well-defined, e.g., histologically characterized NAFLD or NASH.

In summary, we have performed the first Mendelian Randomization analysis to examine the causal role of cannabis use and the risk of fatty liver disease. Our data did not show evidence to support a causal protective effect of cannabis consumption against the development of NAFLD as observed in multiple epidemiological studies. However, our study may be underpowered to verify such a causal relationship. More studies are needed to further understand the broadly observed negative correlation between cannabis consumption and the reduced NAFLD risk. Before this, cautions should be given when considering the medical use of cannabis in NAFLD treatment, as well as the drug development for NAFLD with a focus on cannabis-related pathways.

## Data Availability Statement

Publicly available datasets were analyzed in this study. This data can be found here: https://www.nature.com/articles/s41593-018-0206-1; https://www.nature.com/articles/s41593-019-0416-1; https://www.nature.com/articles/mp2017200?draft=marketing; and https://jamanetwork.com/journals/jamapsychiatry/fullarticle/2504223.

## Author Contributions

XW and ZL analyzed the data and wrote the manuscript. WL conceived the study design and revised the manuscript. All authors contributed to the article and approved the submitted version.

## Conflict of Interest

The authors declare that the research was conducted in the absence of any commercial or financial relationships that could be construed as a potential conflict of interest.

## Footnotes

1 https://sb452.shinyapps.io/power/

2 http://www.r-project.org/
