# Supplementary material for: Does Cannabis Intake Protect Against Non-alcoholic Fatty Liver Disease? A Two-Sample Mendelian Randomization Study
Source: Front Genet. 2020 Aug 14;11:949. doi: 10.3389/fgene.2020.00949 (PMC7457051; doi:10.3389/fgene.2020.00949)
Supplement: Supplementary file 1 [file Table_1.docx]

**Supplementary Table 1. Characteristics of the SNPs used for mendelian randomization analysis.**

| **Phenotype** | **Threshold** | **SNP** | **Original SNP (R^2 with the proxy SNP)** | **Gene** | **Chr** | **Pos (GRCh37)** | **EA/OA** | **EAF** | **SNP-Exposure** | | | **SNP-Outcome (NAFLD)** | | |
| --- | --- | --- | --- | --- | --- | --- | --- | --- | --- | --- | --- | --- | --- | --- |
|  |  |  |  |  |  |  |  |  | **Beta** | **SE** | ***p*** | **Beta** | **SE** | ***p*** |
| Lifetime cannabis  use | *p* < 5E-08 (n=5) | rs2875907 |  | CADM2 | 3 | 85518580 | A/G | 0.352 | 0.071 | 0.009 | 9.38E-17 | 0.030 | 0.044 | 5.04E-01 |
|  |  | rs9919557 |  | NCAM1 | 11 | 112877408 | C/T | 0.386 | 0.055 | 0.009 | 9.94E-11 | 0.009 | 0.043 | 8.29E-01 |
|  |  | rs10499 |  | ATP2A1 | 16 | 28915527 | A/G | 0.651 | 0.053 | 0.009 | 1.13E-09 | 0.026 | 0.044 | 5.56E-01 |
|  |  | rs147644531 | rs9773390 (1.0) | ZNF704 | 8 | 81566155 | A/C | 0.067 | 0.171 | 0.029 | 5.66E-09 | -0.276 | 1.755 | 8.75E-01 |
|  |  | rs17761723 |  | SMG6 | 17 | 2107090 | T/C | 0.346 | 0.047 | 0.009 | 3.24E-08 | 0.038 | 0.045 | 3.89E-01 |
|  | *p* < 1E-05 (n=67) | rs2875907 |  | CADM2 | 3 | 85518580 | A/G | 0.352 | 0.071 | 0.009 | 9.38E-17 | 0.030 | 0.044 | 5.04E-01 |
|  |  | rs9919557 |  | NCAM1 | 11 | 112877408 | C/T | 0.386 | 0.055 | 0.009 | 9.94E-11 | 0.009 | 0.043 | 8.29E-01 |
|  |  | rs10499 |  | ATP2A1 | 16 | 28915527 | A/G | 0.651 | 0.053 | 0.009 | 1.13E-09 | 0.026 | 0.044 | 5.56E-01 |
|  |  | rs147644531 | rs9773390 (1.0) | ZNF704 | 8 | 81566155 | A/C | 0.067 | 0.171 | 0.029 | 5.66E-09 | -0.276 | 1.755 | 8.75E-01 |
|  |  | rs17761723 |  | SMG6 | 17 | 2107090 | T/C | 0.346 | 0.047 | 0.009 | 3.24E-08 | 0.038 | 0.045 | 3.89E-01 |
|  |  | rs466765 |  | REV3L | 6 | 111668494 | A/T | 0.209 | 0.057 | 0.01 | 5.88E-08 | 0.010 | 0.053 | 8.44E-01 |
|  |  | rs1154693 |  | RP11-384F7.1 | 3 | 117804154 | G/A | 0.854 | 0.063 | 0.012 | 6.92E-08 | 0.049 | 0.060 | 4.11E-01 |
|  |  | rs12373990 |  | FAM83F | 22 | 40396028 | T/C | 0.117 | 0.067 | 0.013 | 1.99E-07 | -0.087 | 0.066 | 1.88E-01 |
|  |  | rs6827295 |  | PCDH7 | 4 | 31135876 | G/T | 0.714 | 0.047 | 0.009 | 2.49E-07 | -0.075 | 0.047 | 1.13E-01 |
|  |  | rs12211611 |  | RAB23 | 6 | 57065443 | G/C | 0.808 | 0.054 | 0.01 | 2.55E-07 | 0.049 | 0.054 | 3.66E-01 |
|  |  | rs520731 | rs1066339 (0.94) | RP1-253B10.2 | 6 | 87465749 | T/C | 0.168 | 0.147 | 0.029 | 2.72E-07 | 0.076 | 0.076 | 3.16E-01 |
|  |  | rs9972422 |  | RNU2-3P | 15 | 96303368 | G/A | 0.291 | 0.046 | 0.009 | 2.75E-07 | 0.041 | 0.047 | 3.79E-01 |
|  |  | rs437021 |  | NFIA | 1 | 61738270 | C/T | 0.541 | 0.042 | 0.008 | 2.77E-07 | 0.043 | 0.042 | 3.10E-01 |
|  |  | rs576076 |  | SLC22A12 | 11 | 64360623 | A/G | 0.253 | 0.048 | 0.009 | 4.43E-07 | -0.073 | 0.049 | 1.35E-01 |
|  |  | rs114212469 |  | RP11-419L4.1 | 4 | 130307649 | T/C | 0.021 | 0.158 | 0.032 | 6.23E-07 | -0.306 | 0.148 | 3.83E-02 |
|  |  | rs1808579 |  | C18orf8 | 18 | 21104888 | T/C | 0.479 | 0.041 | 0.008 | 6.80E-07 | -0.090 | 0.042 | 3.34E-02 |
|  |  | rs9435794 |  | ACTL8 | 1 | 18131916 | C/T | 0.289 | 0.046 | 0.009 | 9.20E-07 | 0.005 | 0.047 | 9.09E-01 |
|  |  | rs7871607 |  | RASEF | 9 | 85546352 | G/T | 0.012 | 0.198 | 0.04 | 9.21E-07 | -0.257 | 0.211 | 2.23E-01 |
|  |  | rs205723 |  | RP11-138A9.2 | 7 | 130621451 | A/G | 0.414 | 0.041 | 0.008 | 1.03E-06 | 0.007 | 0.043 | 8.79E-01 |
|  |  | rs60369116 |  | CYTH3 | 7 | 6225044 | G/C | 0.975 | 0.143 | 0.03 | 1.24E-06 | -0.116 | 0.139 | 4.05E-01 |
|  |  | rs11902472 |  | AC104820.2 | 2 | 181990859 | A/G | 0.617 | 0.041 | 0.008 | 1.31E-06 | 0.028 | 0.044 | 5.23E-01 |
|  |  | rs4377758 |  | RP11-486E2.1 | 6 | 81509505 | G/T | 0.056 | 0.094 | 0.02 | 1.34E-06 | -0.027 | 0.092 | 7.69E-01 |
|  |  | rs79294243 |  | RNU6-715P | 2 | 148273120 | C/T | 0.044 | 0.099 | 0.021 | 1.64E-06 | -0.067 | 0.103 | 5.16E-01 |
|  |  | rs10849982 |  | RP3-462E2.5 | 12 | 112335539 | G/A | 0.828 | 0.052 | 0.011 | 1.76E-06 | 0.112 | 0.056 | 4.52E-02 |
|  |  | rs2059730 |  | THSD7B | 2 | 137562613 | G/A | 0.669 | 0.042 | 0.009 | 1.85E-06 | -0.037 | 0.046 | 4.25E-01 |
|  |  | rs11209802 |  | RP11-399E6.1 | 1 | 41756826 | C/T | 0.679 | 0.042 | 0.009 | 1.96E-06 | 0.036 | 0.045 | 4.22E-01 |
|  |  | rs146752096 |  | AC007679.3 | 2 | 206851318 | T/G | 0.086 | 0.069 | 0.015 | 1.96E-06 | 0.032 | 0.077 | 6.75E-01 |
|  |  | rs1012534 |  | ARID1B | 6 | 157209663 | A/G | 0.566 | 0.039 | 0.008 | 2.25E-06 | 0.036 | 0.043 | 3.99E-01 |
|  |  | rs1885331 |  | BAI3 | 6 | 70007550 | T/G | 0.751 | 0.045 | 0.01 | 2.33E-06 | 0.076 | 0.049 | 1.19E-01 |
|  |  | rs2305758 |  | HAUS8 | 19 | 17163661 | T/C | 0.28 | 0.043 | 0.009 | 2.70E-06 | -0.020 | 0.047 | 6.76E-01 |
|  |  | rs78698099 |  | CACNG3 | 16 | 24367577 | G/A | 0.95 | 0.101 | 0.022 | 2.78E-06 | 0.040 | 0.097 | 6.82E-01 |
|  |  | rs13123620 |  | LDB2 | 4 | 16915220 | A/G | 0.591 | 0.039 | 0.008 | 3.20E-06 | -0.018 | 0.043 | 6.73E-01 |
|  |  | rs79777905 |  | MSRA | 8 | 10078246 | G/A | 0.982 | 0.172 | 0.037 | 3.32E-06 | 0.134 | 0.160 | 4.00E-01 |
|  |  | rs9855698 |  | GRM7 | 3 | 7371861 | G/C | 0.14 | 0.056 | 0.012 | 3.73E-06 | 0.009 | 0.061 | 8.80E-01 |
|  |  | rs1587858 |  | CTC-281M20.1 | 5 | 174554702 | C/T | 0.301 | 0.042 | 0.009 | 3.79E-06 | 0.004 | 0.046 | 9.24E-01 |
|  |  | rs1503510 |  | RP11-158J3.2 | 5 | 63067793 | C/T | 0.65 | 0.04 | 0.009 | 3.94E-06 | 0.009 | 0.044 | 8.32E-01 |
|  |  | rs9655332 |  | PDE1C | 7 | 32004279 | T/G | 0.423 | 0.067 | 0.015 | 3.95E-06 | -0.273 | 1.192 | 8.19E-01 |
|  |  | rs61942416 |  | RP11-417L19.2 | 12 | 132356197 | A/G | 0.078 | 0.077 | 0.017 | 3.99E-06 | -0.040 | 0.081 | 6.21E-01 |
|  |  | rs701802 |  | ARHGAP19-SLIT1 | 10 | 98948238 | G/A | 0.64 | 0.041 | 0.009 | 4.14E-06 | -0.071 | 0.044 | 1.09E-01 |
|  |  | rs7513688 |  | KLHL21 | 1 | 6652745 | G/A | 0.642 | 0.039 | 0.009 | 4.48E-06 | 0.031 | 0.044 | 4.79E-01 |
|  |  | rs2086512 |  | RP3-453D15.1 | 6 | 99011501 | A/G | 0.109 | 0.06 | 0.013 | 4.55E-06 | 0.044 | 0.069 | 5.27E-01 |
|  |  | rs73262787 |  | APOBEC1 | 12 | 7813787 | G/A | 0.947 | 0.086 | 0.019 | 4.63E-06 | -0.067 | 0.095 | 4.80E-01 |
|  |  | rs6948053 |  | HIP1 | 7 | 75237295 | G/A | 0.056 | 0.086 | 0.019 | 4.66E-06 | -0.011 | 0.095 | 9.08E-01 |
|  |  | rs830133 | rs830147 (1.0) | SLC8A2 | 19 | 47974578 | G/A | 0.947 | 0.17 | 0.037 | 4.73E-06 | 0.090 | 0.098 | 3.55E-01 |
|  |  | rs4837004 |  | GOLGA1 | 9 | 127663316 | C/T | 0.34 | 0.039 | 0.009 | 4.79E-06 | -0.016 | 0.045 | 7.14E-01 |
|  |  | rs4147187 |  | RP11-308N19.4 | 9 | 109418599 | T/C | 0.021 | 0.143 | 0.031 | 4.95E-06 | 0.022 | 0.145 | 8.78E-01 |
|  |  | rs149434117 |  | SEC22C | 3 | 42592540 | T/C | 0.983 | 0.164 | 0.036 | 4.96E-06 | 0.167 | 0.161 | 2.98E-01 |
|  |  | rs2335349 |  | IPMKP1 | 13 | 23404504 | T/C | 0.481 | 0.038 | 0.008 | 5.01E-06 | -0.040 | 0.042 | 3.50E-01 |
|  |  | rs12949052 |  | RP11-159D12.10 | 17 | 56142010 | A/T | 0.926 | 0.073 | 0.016 | 5.10E-06 | 0.059 | 0.081 | 4.66E-01 |
|  |  | rs62638743 |  | COL5A3 | 19 | 10088360 | A/G | 0.03 | 0.116 | 0.025 | 5.10E-06 | -0.104 | 0.126 | 4.07E-01 |
|  |  | rs11749751 |  | RP11-120B7.1 | 5 | 107901554 | G/A | 0.205 | 0.047 | 0.01 | 5.18E-06 | -0.004 | 0.053 | 9.38E-01 |
|  |  | rs6047198 |  | RP5-1177M21.1 | 20 | 20989003 | C/T | 0.753 | 0.043 | 0.01 | 5.41E-06 | 0.022 | 0.049 | 6.48E-01 |
|  |  | rs11724871 |  | MIR4801 | 4 | 37186348 | C/A | 0.467 | 0.038 | 0.008 | 5.77E-06 | 0.077 | 0.042 | 6.93E-02 |
|  |  | rs7969834 |  | ANO2 | 12 | 5901663 | G/A | 0.273 | 0.041 | 0.009 | 6.58E-06 | -0.046 | 0.048 | 3.33E-01 |
|  |  | rs10012797 |  | RGS12 | 4 | 3385176 | A/T | 0.917 | 0.068 | 0.015 | 6.63E-06 | 0.007 | 0.077 | 9.23E-01 |
|  |  | rs143529057 |  | CTC-340A15.2 | 5 | 164566490 | C/T | 0.989 | 0.204 | 0.045 | 6.89E-06 | 0.082 | 0.208 | 6.93E-01 |
|  |  | rs17294232 |  | AC064834.1 | 2 | 196333052 | A/C | 0.554 | 0.038 | 0.008 | 7.11E-06 | -0.088 | 0.043 | 4.03E-02 |
|  |  | rs61997596 |  | TDRD9 | 14 | 104511206 | A/G | 0.186 | 0.048 | 0.011 | 7.11E-06 | -0.004 | 0.054 | 9.38E-01 |
|  |  | rs79563551 |  | TUBB4BP5 | 4 | 122395365 | C/T | 0.978 | 0.143 | 0.032 | 7.50E-06 | 0.112 | 0.143 | 4.33E-01 |
|  |  | rs17481131 |  | SOX5 | 12 | 24172260 | T/C | 0.793 | 0.045 | 0.01 | 8.06E-06 | 0.002 | 0.052 | 9.71E-01 |
|  |  | rs139621111 |  | ASNS | 7 | 97443791 | C/A | 0.196 | 0.088 | 0.02 | 8.15E-06 | -0.058 | 0.052 | 2.59E-01 |
|  |  | rs7670670 |  | GUCY1A3 | 4 | 156573674 | T/C | 0.224 | 0.044 | 0.01 | 8.18E-06 | 0.001 | 0.051 | 9.92E-01 |
|  |  | rs80144387 |  | AC131097.3 | 2 | 242839021 | T/G | 0.07 | 0.086 | 0.019 | 8.76E-06 | 0.016 | 0.083 | 8.49E-01 |
|  |  | rs2049824 |  | RN7SKP226 | 8 | 129289556 | C/A | 0.467 | 0.037 | 0.008 | 9.08E-06 | -0.060 | 0.042 | 1.58E-01 |
|  |  | rs10849767 |  | RPL11P5 | 12 | 121034286 | T/C | 0.334 | 0.04 | 0.009 | 9.38E-06 | 0.010 | 0.045 | 8.23E-01 |
|  |  | rs72798040 |  | RP11-429P3.5 | 16 | 50149693 | T/C | 0.901 | 0.062 | 0.014 | 9.40E-06 | -0.002 | 0.071 | 9.78E-01 |
|  |  | rs4445597 |  | RP11-379J13.2 | 11 | 97854975 | T/G | 0.105 | 0.06 | 0.014 | 9.86E-06 | -0.045 | 0.069 | 5.16E-01 |
| Cannabis use disorder | *p* < 5E-08 (n=1) | rs56372821 |  | *CHRNA2* | 8 | 27436500 | A/G | 0.163 | -0.22 | 0.032 | 9.09E-13 | -0.034 | 0.058 | 5.60E-01 |
| Cannabis use dependence | *p* < 5E-08 (n=1) | rs1409568 |  | H3K4me1 /H3K427ac | 10 | 120630785 | T/C | 0.941 | -0.50 | 0.09 | 3.95E-08 | -0.168 | 0.090 | 6.34E-01 |
| Cannabis use criterion count | *p* < 5E-08 (n=2) | rs143244591 |  | *RP11-206M11.*7 | 3 | 149013935 | G/A | 0.996 | 0.54 | 0.087 | 4.32E-10 | 0.059 | 0.373 | 8.75E-01 |
|  |  | rs77378271 |  | *CSMD1* | 8 | 3073489 | A/G | 0.948 | 0.29 | 0.052 | 2.13E-08 | -0.070 | 0.097 | 4.67E-01 |

Gene: overlapped or nearest gene; EA: effect allele; OA: other allele; EAF: effect allele frequency; Beta: coefficient of the effect allele; SE: standard error of the coefficient
